# Long-term health of women with genetic POI due to FSH-resistant ovaries

**DOI:** 10.1530/EC-19-0244

**Published:** 2019-09-09

**Authors:** Kaisu Luiro, Kristiina Aittomäki, Pekka Jousilahti, Juha S Tapanainen

**Affiliations:** 1 Department of Obstetrics and Gynecology, Reproductive Medicine Unit, Helsinki University Hospital and University of Helsinki, Helsinki, Finland; 2 Department of Medical Genetics, Helsinki University Hospital, Helsinki, Finland; 3 Department of Public Health Solutions, National Institute for Health and Welfare, Helsinki, Finland; 4 Department of Obstetrics and Gynecology, University of Oulu and Oulu University Hospital, Medical Research Center, PEDEGO Research Unit, Oulu, Finland

**Keywords:** POI, primary ovarian insufficiency, primary amenorrhea, FSH receptor, osteopenia

## Abstract

**Objective:**

To study the use of hormone therapy (HT), morbidity and reproductive outcomes of women with primary ovarian insufficiency (POI) due to FSH-resistant ovaries (FSHRO).

**Design:**

A prospective follow-up study in a university-based tertiary clinic setting.

**Methods:**

Twenty-six women with an inactivating A189V FSH receptor mutation were investigated by means of a health questionnaire and clinical examination. Twenty-two returned the health questionnaire and 14 were clinically examined. Main outcome measures in the health questionnaire were reported as HT, morbidity, medication and infertility treatment outcomes. In the clinical study, risk factors for cardiovascular disease (CVD) and metabolic syndrome (MetS) were compared to age-matched controls from a national population survey (FINRISK). Average number of controls was 326 per FSHRO subject (range 178–430). Bone mineral density and whole-body composition were analyzed with DXA. Psychological and sexual well-being was assessed with Beck Depression Inventory (BDI21), Generalized Anxiety Disorder 7 (GAD-7) and Female Sexual Function Index (FSFI) questionnaires.

**Results:**

HT was initiated late (median 18 years of age) compared with normal puberty and the median time of use was shorter (20–22 years) than the normal fertile period. Osteopenia was detected in 9/14 of the FSHRO women despite HT. No major risk factors for CVD or diabetes were found.

**Conclusions:**

HT of 20 years seems to be associated with a similar cardiovascular and metabolic risk factor profile as in the population control group. However, optimal bone health may require an early-onset and longer period of HT, which would better correspond to the natural fertile period.

## Introduction

Primary ovarian insufficiency (POI) is a clinical condition defined by loss of ovarian activity before the age of 40 years (1). Characteristic features include menstrual disturbances (amenorrhea or oligomenorrhea), elevated levels of FSH and low estradiol and anti-Müllerian hormone (AMH) levels. The etiology of POI is heterogeneous with a significant genetic contribution (2). Ten to fifteen percent of POI patients have an affected first-degree relative (3). Autosomal, recessively inherited FSH receptor mutations underlie a genetic form of POI named FSH-resistant ovaries syndrome (FSHRO) (4). The incidence has been estimated to be 1:10,000-1:30,000 in Finland. Since 1995, when the mutation was discovered, altogether 40 patients have been diagnosed, that is, one affected girl is born every year. This inactivating Ala189Val mutation was the first genetic form of POI that was functionally characterized in detail. The clinical phenotype of FSHRO patients has also been carefully analyzed (5). Compared with patients with a POI of unknown origin, FSHRO patients were shorter and pubertal development was variable but not significantly different. Gonadotropin levels were postmenopausally high and those of estradiol at the low end of the normal range. Levels of AMH were normal in contrast to other types of POI (6). Notably, primordial, primary and, in two cases, preantral follicles were histologically detected in six of the eight FSHRO patients. However, in the absence of a functional FSH receptor, follicular maturation did not occur, resulting in primary infertility. Infertility in FSHRO women has been effectively managed by ovum donation, indicating that FSH function is not essential in achieving successful pregnancy using donated oocytes (7).

Adverse long-term health outcomes of POI have been indicated in several epidemiological and observational studies (8, 9, 10, 11). POI is associated with shortened life expectancy and increased mortality mainly due to CVD, osteoporosis and fractures. In addition, impairment of cognitive functions, dementia, Parkinsonism, and reduced sexual function and psychological well-being have been reported (12). Most of the evidence comes from observational studies of women who have undergone iatrogenic, surgical menopause in the form of bilateral oophorectomy. Long-term health implications of non-iatrogenic POI may be different due to the more gradual and fluctuating course of the disease.

The inactivating FSH receptor mutation was discovered 20 years ago, but its long-term effects on health and morbidity, and the effect of estrogen hormone therapy (HT) have not been thoroughly investigated. There is evidence that some of the adverse events related to POI may be prevented by estrogen HT. Currently, no prospective, long-term studies have been carried out to investigate the influence of HT in POI. In this study, we assessed the health and morbidity of an FSHRO cohort by means of a questionnaire, followed by detailed clinical examination.

## Materials and methods

### Subjects

Altogether 40 FSHRO patients with a confirmed inactivating (A189V) FSH receptor (*FSHR*) mutation have been identified in Finland since 1995. We could locate 26 women, who were sent a questionnaire regarding general health, morbidity and fertility. They were also asked to participate in an extensive clinical examination. Twenty-two patients were willing to take part in the clinical study, but for practical reasons (moving abroad, ongoing IVF, new-born infant, another simultaneous health examination, transport or timing difficulties) 14 women were clinically examined, 12 at Helsinki University Hospital and 2 at Oulu University Hospital. All subjects gave written informed consent. The study protocol was approved by the Hospital District of Helsinki and Uusimaa Ethics Committee for Gynecology and Obstetrics, Pediatrics and Psychiatry, and the Ethics Committee of Oulu University Hospital.

The control group was formed from participants in a national FINRISK 2007 Study. The FINRISK Study is a national population risk factor monitoring survey carried out at 5-year intervals by the National Institute for Health and Welfare (NIHW). For each survey, an independent random sample is drawn from the national population register stratified by sex and 10-year age group from the population aged 25–74 years separately for each survey area (13). Altogether, 3013 women were examined in the FINRISK 2007 Study and for each FSHRO subject an average of 326 (range 178-430) age-matched (±2 years) control women were found. The data are collected in the NIHW Biobank and we obtained it in the following format: number of available control subjects based on the age criteria, mean and standard deviation for each parameter. These means formed the control group for a given parameter. This study was approved by the Ethics Committee of the NIHW.

### Questionnaires

The questionnaire included detailed questions regarding the use of HT, infertility treatments and their outcome(s), bone density measurements and incidence of osteoporosis, and cardiovascular symptoms and disease. The patients were also asked to report any malignancies, operations, and neurological, psychological, respiratory, gastrointestinal, cutaneous, or allergy symptoms.

To assess psychological and sexual well-being, the following questionnaires were used: Beck Depression Inventory (BDI) (14), Generalized Anxiety Disorder 7 (GAD-7) (15), Female Sexual Function Index (FSFI) (16).

### Measurements for lipid profile

For the FSHRO cohort, venous blood samples after overnight fasting were collected for the analysis of total cholesterol, high-density lipoprotein cholesterol (HDL-c), low-density lipoprotein cholesterol (LDL-c) and triglycerides. For the FSHRO group measurements for lipids were performed at Helsinki University Hospital laboratory (HUSLAB). For the control group assays were performed at the Laboratory of Analytical Biochemistry at the Center for Health and Welfare.

### Oral glucose tolerance test (OGTT)

Oral glucose tolerance tests with 75 g of glucose were performed after overnight fasting for 12 h. Venous blood samples were drawn to assay levels of glucose and insulin at baseline, and at 30, 60 and 120 min in the FSHRO cohort and at baseline and 120 min in the control group. For the FSHRO group plasma glucose and insulin measurements were performed immediately after blood sampling at HUSLAB. For the control group assays were performed at the Laboratory of Analytical Biochemistry at the Center for Health and Welfare. The glucose tolerance test results were analyzed according to WHO criteria, which defines impaired fasting glucose (IFG) as fasting glucose concentrations of 6.1–6.9 mmol/L and impaired glucose tolerance (IGT) as 2-h glucose concentrations of 7.8–11.0 (17). In addition, concentrations of glycosylated hemoglobin (HbA1c) were determined in the overnight fasting blood samples.

### Dual energy X-ray absorptiometry (DXA)

DXA was performed using a Lunar Prodigy Advance device (GE Healthcare). Adult whole-body software was used for data acquisition and analysis. All patients were examined in a supine position. Total body and regional body fat and lean masses were assessed. Total body fat (TBF), total body lean mass (TBL), android fat (AF), and gynoid fat (GF) were expressed as a percentage (%) of total body mass. The AF-to-GF ratio (A/G) and TBF-to-TBL ratio (TBF/TBL) were calculated. The android region was considered to extend from the pubis up to the fifth of an ideal line extending from the pubis to the jugulum. The gynoid region is considered to be delimited by the upper greater trochanters, and a boundary defined at a distance of twice the height of the android region. Both AF and GF were expressed as percentage of the TBF.

### Gynecological examination and transvaginal ultrasonography

Standard pelvic examination and transvaginal ultrasonography were performed by a trained gynecologist (K.L.). Transvaginal ultrasonography was performed with GE Voluson E8, D00309 equipment. The ovaries were visualized in three dimensions and their volumes were calculated. Antral follicle count was performed when applicable.

### Statistical analysis

When comparing distributions of variables in the FSHRO women who completed both the health questionnaire study and the clinical examination to the FSHRO women who only returned the health questionnaire, the Mann–Whitney *U* test was used for continuous variables.

When comparing distributions of variables in the FSHRO cohort and the FINRISK control group, conditional logistic regression analysis was used. This method was selected due to the small number of subjects and the age-matched control group, where a normal distribution could not be assumed. Two-sided *P* values lower than 0.05 were considered statistically significant. Statistical analyses were performed using IBM SPSS Statistics 24 software.

## Results

### Health questionnaire

A cohort of 26 women with a previously confirmed inactivating FSH receptor (*FSHR*) mutation was asked to participate in a questionnaire study regarding HT use, morbidity and fertility. Twenty-two women (22/26) aged 28–70 years (median age 51 years) completed and returned the questionnaire. The use of HT is summarized in Table 1. All patients had been on HT. In addition, three women were using local vaginal estrogen therapy at the time of the investigation. The median starting age for the use of HT was 18 years (range 15–45), and the median time of use was 22 years (range 1–35).

**Table 1:** Use of hormone therapy in the FSHRO cohort as reported in the health questionnaire.

		Range
HT use (*n*)	22/22	N.A.
HT current use (*n*)	10/22	N.A.
Age at HT onset, median	18	15–45
HT use (years), median	22	1–40
HT use below age 50 (years), median	19.5	3–33

HT, hormone therapy.

**Figure 1:**
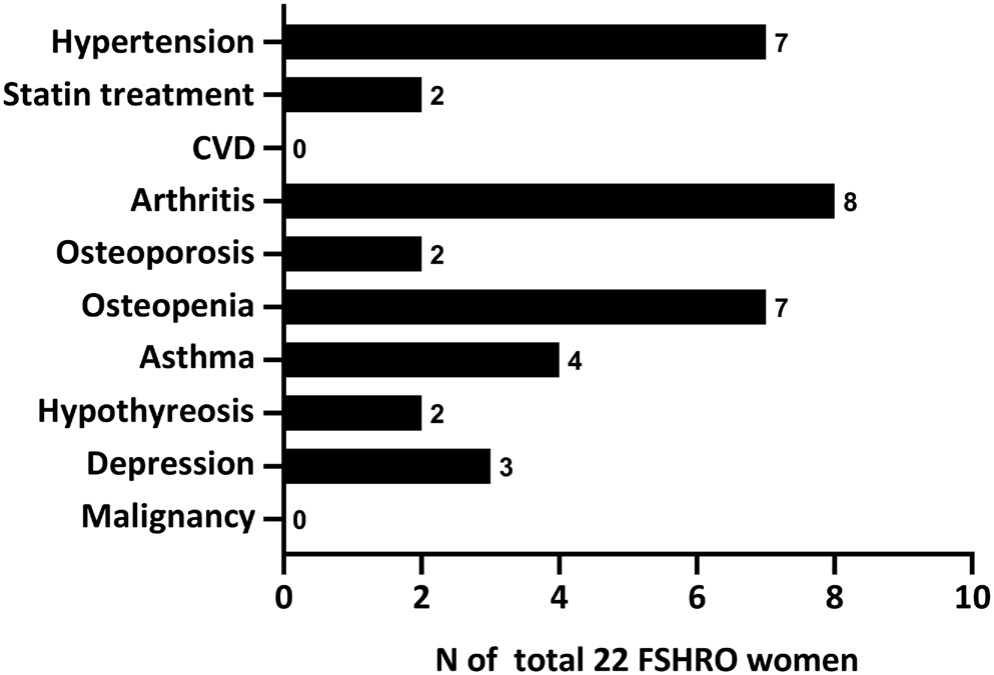
Comorbidities in the FSHRO cohort as reported in the health questionnaire. CVD, cardiovascular disease; PPI, proton-pump inhibitor.

Reported comorbidities and medications are shown in Fig. 1. No coronary heart disease, myocardial infarction or other serious cardiovascular events were reported. This was lower than expected, as in a national survey self-reported coronary heart disease or previous myocardial infarction in women has ranged from 0.7 to 2.2% (18). Reported frequency of statin treatment (2/22; 9.1%) was comparable to the national frequency of 12.4% reimbursed statin treatments in whole population (National Registry for Medication Reimbursement, https://www.kela.fi/tilastotietokanta-kelasto_sisallysluettelo#Sairastaminen, accessed 3.12.2018). The median duration of HT in women with hypertension was 28 years versus 20 years in women without hypertension. However, the median age of women with hypertension (53 years) was also 3 years older than women without hypertension.

Eight women (8/22) reported arthritis, and one had undergone bilateral total hip arthroplasty as a result of arthritis. A majority of the FSHRO women (16/22) had undergone bone mineral density assessment (DXA). Two women (2/22) reported osteoporosis, seven (7/22) had osteopenia, three (3/22) had normal bone mineral density, and four (4/22) had either lost their DXA report or did not remember the result. There was no significant relationship between the duration of HT and osteoporosis or osteopenia.

Four women (4/22, 18.2%) were diagnosed with asthma, which is slightly more compared to the self-reported 11.6% in a national health survey of women of 30–75 years of age (19). Three (3/22, 13.6%) women had suffered from mood disorder and had been treated with antidepressants and psychotherapy, which was more frequent compared to the reported national prevalence of depressive disorders (8.2%) (20). At the time of this study, one of FSHRO subjects was using antidepressant medication.

Fourteen FSHRO women (14/22) had undergone infertility treatments via IVF with donated oocytes, as summarized in Supplementary Fig. 1 (see section on supplementary data given at the end of this article). Altogether 15 live births had been achieved. During pregnancy, two women had had pre-eclampsia and one had suffered pulmonary embolism. One child, whose mother had anti-nuclear SSA autoantibodies (anti-Sjögren’s-syndrome-related antigen A), had a total AV block. Otherwise the children were healthy, and all except one were full term. Ten women delivered vaginally, four women had had an elective cesarean section and one woman had had an emergency cesarean section.

### Clinical study

Fourteen FSHRO women took part in the clinical part of the study. No statistically significant differences were found in the demographic characteristics of the FSHRO women who completed both the health questionnaire and clinical examination and the FSHRO women who only returned the health questionnaire (Supplementary Table 1). The demographic data of the clinical study cohort, as well as lipid and glucose metabolism profiles, were compared with those in a national population risk factor monitoring study (FINRISK 2007) (18) shown in Table 2. The characteristics of the FSHRO study cohort were comparable to those of the age-matched controls. One of the FSHRO patients (1/22, 7.1%) had medication for elevated blood pressure, whereas 27.4% of the FINRISK 2007 subjects had either BP over 140/90 mmHg or medication. Three of the FSHRO patients were smokers (3/22, 21.4%), which is markedly higher incidence compared to the control group (13.1%).

**Table 2:** Characteristics of the FSHRO clinical study cohort and the control group from the FINRISK 2007 Study.

	FSHRO			FINRISKI 2007 control		
		Range	±s.d.		Range	±s.d.
*n*	14			326^a^	178–430	
Age (y), mean	50.2	31–70			±2 years	
Height (m) mean	1.62	1.54–1.73	5.7	1.60		0.06
BMI (kg/m^2^), mean	26.1	21.2–42.1	5.4	26.7		5.3
WHR, mean	0.8	0.77–0.98	0.07	0.85		0.06
Systolic BP (mmHg), mean	126	106–151	13	126		17
Diastolic BP (mmHg), mean	79	69–90	7	78		10
Smoking (%)	21.4%			13.1%		

^a^For each FSHRO subject an average of 326 age-matched controls were found (range 178–430 controls). In total, 3013 women were examined in the FINRISK 2007 Study.

BMI, body mass index; BP, blood pressure; HT, hormone therapy; WHR, waist-to-hip ratio.

In the clinical study cohort, all FSHRO patients had been on HT, and six (6/14) were currently on it. Corresponding to the results of the questionnaire study, the median time of HT use was 20 years, which is shorter than the normal fertile period. This was mainly a result of delayed diagnosis, problems with renewing the prescription at GP (after an initial specialist prescription) or lack of motivation to undergo HT. None of the FSHRO women reported any typical vasomotor climacteric symptoms such as hot flushes or night sweats, which could diminish compliance to HT.

In gynecological examination, vaginal atrophy (thin, pale and dry appearance) was detected in five patients. One patient had spotting and a benign cervical polyp was removed. Twelve patients had small streak ovaries. In two patients (aged 31 and 36) antral follicles were detected.

### Lipid profile

**Figure 2:**
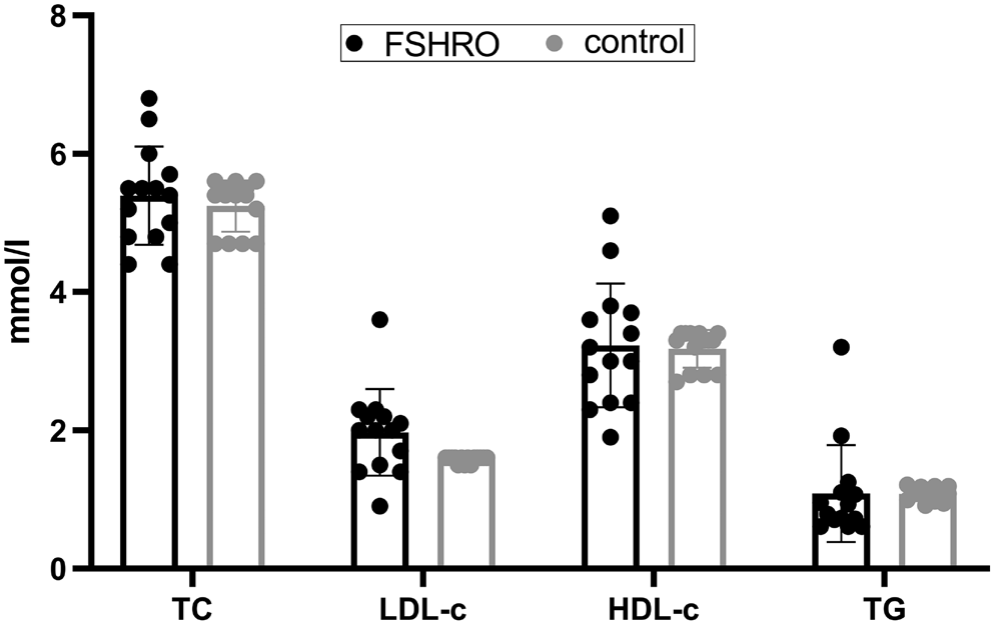
Lipid profile of the FSHRO and control groups. Data points for the control group represent the mean of all age-matched controls for each FSHRO subject from the FINRISK Study. Box indicates mean, bars indicate ±s.d. HDL-c, high-density lipoprotein cholesterol; LDL-c, low-density lipoprotein cholesterol; TC, total cholesterol; TG, triglycerides.

To evaluate the risk of CVD, the lipid profile of the FSHRO cohort was examined. The results are shown in Fig. 2 in comparison with the control group from the national FINRISK 2007 Study. No significant differences in the lipid measurements were detected between the FSHRO cohort and the age-matched control group (*P* < 0.05). It has been suggested that an LDL-c/ HDL-c ratio is a better risk indicator for CVD than individual parameters (21). In the FSHRO cohort, the LDL-c/HDL-c ratio was 1.8 (0.99–5.43). When examining women over 50 years of age (*n*=9), the duration of HT did not alter the LDL-c/HDL-c ratio significantly. In addition, there was no significant relationship with current HT use and the LD-cL/HDL-c ratio in this cohort.

### Oral glucose test (OGTT)

**Figure 3:**
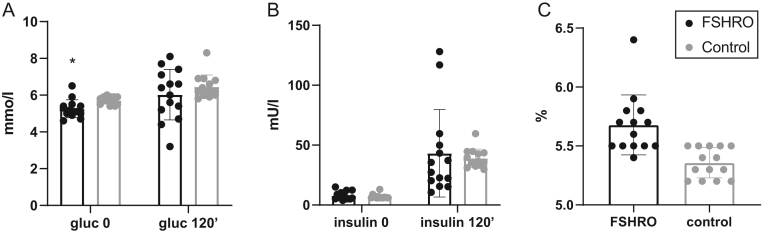
Glucose metabolism in FSHRO and control groups. (A) Mean glucose levels in OGTT. Gluc 0’, fasting glucose; gluc 120’, glucose at 120 min. *P = 0.026. (B) Mean insulin levels in OGTT. Insulin 0’, fasting insulin; insulin 120’, insulin at 120 min. (C) Mean levels of glycosylated hemoglobin (HbA1c). No statistically significant difference between the groups (*P* = 0.136). Data points for the control group represent the mean of all age-matched controls for each FSHRO subject from the FINRISK Study. Box indicates mean, bar indicates ±s.d.

According to WHO criteria, no cases of diabetes was found in the FSHRO cohort. One person had IFG with a plasma glucose level of 6.5 mmol/L. No IGT was detected. The mean level of HbA1c, reflecting the 3-month average of plasma glucose concentrations, was also normal (38.6±1.6 mmol/L) corresponding to 5.6%. The parameters related to glucose metabolism are shown in Fig. 3A, B and C in comparison with the age-matched control group from the FINRISK 2007 Study. The mean level of fasting glucose in the FSHRO cohort was 5.5 mmol/L, which was statistically significantly lower than the control group (5.7 mmol/L, *P*=0.026). No other significant differences were found between the groups.

### DXA

DXA with whole-body composition analysis was performed. The mean amount of TBF was 39.3 kg (range 31.7–47.1) and the total body lean mass (TBL) was 64.2 kg (range 56.0–72.3) resulting in a TBF/TBL ratio of 0.60 (range 0.44–1.16). To predict the risk for MetS and CVD, the android/GF ratio (A/G ratio) was determined. A ratio over 0.8 is considered to be a risk factor. The mean A/G ratio was elevated 1.0 (range 0.73–1.16). With regard to the duration of HT, there was no significant difference in the A/G ratio in subgroups that had been on HT for over or under 20 years (*P*=0.343).

Bone mineral density (BMD) was measured at the lumbar spine (L1-4) and femur neck. One woman had a T-score of −2.8 in her lumbar spine indicating osteoporosis. This 37-year-old patient had used (and was still using) HT for 20 years, but had also smoked daily for 20 years. Eight of the FSHRO subjects (64.3%) had T-scores indicating osteopenia (−1.0 to −2.5). Six of the osteopenic women were not currently using HT. However, as a result of small sample size, we found no significant relationship between current HT use and osteopenia. Among the women over 50 years of age (*n*=9), seven had osteopenia. Five of these women had used HT for over 20 years, and we found no significant relationship between the duration of the use of HT and osteopenia, again most likely due to the small sample size.

### Psychological and sexual well-being

Primary amenorrhea has been reported to have adverse effects on psychological and sexual well-being. The Beck Depression Inventory (BDI) was used to assess the incidence and severity of depression. The median score was 3.5 (range 0–27) indicating no clinical depression. Two patients had elevated scores indicating moderate depression. No statistically significant association with HT use and BDI score was found. To assess generalized anxiety disorder in the study cohort, GAD-7 was utilized. Scores over 10 indicate GAD. The median score was 0.5 (range 0–9) indicating no detectable GAD in the FSHRO cohort. Accordingly, no statically significant association with HT and GAD-7 score was found. To investigate female sexual function in the FSHRO cohort, a multidimensional, self-reported questionnaire was used (FSFI). This includes separate assessment of six different domains of sexual function: desire, arousal, lubrication, orgasm, satisfaction and pain. Each domain is scored on a scale of 0–5, with higher scores indicating better function. One subject declined answering the FSFI. The mean FSFI score was 17.5/36 (range 1.5–32.8). Of the FSHRO subjects, 9/13 scored under 73% of the maximum score, indicating a risk of sexual dysfunction. Moreover, 4/13 women scored under 33% of the maximum score indicating a high risk of sexual dysfunction. Twelve of the FSHRO women were in a long-term relationship (marriage, common law marriage, registered partnership). Relationship status did not have a significant impact on sexual well-being in this cohort. The presence of vaginal atrophy did not affect the FSFI score. Only one person with vaginal atrophy had an FSFI score indicating a risk of sexual dysfunction. No statistically significant association was found with current HT use or duration of HT use and total the FSFI score or its six separate dimensions.

## Discussion

The aim of this study was to elucidate the long-term health, morbidity and reproductive outcomes of women with the FSHRO syndrome and investigate the effect of HT. FSHRO is a recessively inherited single gene disorder causing genetic POI. Due to its explicit etiology without confounding environmental factors, it can serve as a model disease for POI. In the FSHRO cohort, a majority of the women had osteopenia and one person had osteoporosis despite HT use. However, the initiation of HT was late compared to the onset of normal puberty. In addition, the median use of HT, 20–22 years, provided a significantly shorter period of exposure to estrogen than the normal fertile period. No major cardiovascular or metabolic disease risk factors or morbidity were detected in the FSHRO cohort compared to the age-matched control group.

This study revealed high total body fat weight (TBF) and an increased android-to-gynoid (A/G) ratio in the FSHRO women, reflecting elevated risks of CVD and MetS. However, when compared with the age-matched women of the FINRISK 2007 population risk factor monitoring survey, no elevated risk for CVDs was found in the FSHRO women. This may in part be a result of the small number of FSHRO subjects, as women with POI have previously been reported to have greater risks for coronary heart disease (CHD), cardiovascular mortality, and all-cause mortality, but no association with stroke (22). Usually this is attributed to the shortened length of estrogen exposure, but it has also been suggested that spontaneous POI is a first step on a causal pathway to multiorgan dysfunction. Due to effective lifestyle intervention programs in the past 35 years, Finland has experienced a remarkable change from the world’s highest coronary heart disease incidence to an 80% decrease in CHD mortality (13), which may in part explain our findings. In addition, risk factors for CVD and MetS may increase as the FSHRO cohort ages, and a follow-up study will enlighten this.

In the FSHRO cohort, eight patients (64,3%) had osteopenia and one person had osteoporosis despite HT use. However, the median use of HT, 20–22 years, provided a significantly shorter period of exposure to estrogen than the normal fertile period, and it seems that it is not adequate in preventing bone loss. POI is a well-established risk factor for osteopenia, osteoporosis and fracture, especially if hypoestrogenism begins before peak bone mass has been reached (23, 24, 25). The risk of fracture is 1.5- to 3-fold higher in women with menopause before the age of 45 years compared with women with menopause at normal average of 50–51 years of age (26, 27). HT has been shown to effectively reduce this risk and it should be the first-line treatment of osteopenia/osteoporosis in women with POI (28, 29). Six of the osteopenic FSHRO women were not currently using HT and five of them were over 50 years of age. The effect of HT withdrawal on bone loss has been mostly studied in postmenopausal women and the results are controversial. Most studies indicate that discontinuation of HT is followed by accelerated bone loss, but long-term use of HT (over 10–15 years) protects women from bone loss (30). We were not able to demonstrate a significant relationship between current HT use and bone loss in the FSHRO cohort. In contrast, FSH has been shown have a direct stimulatory effect on osteoclast-mediated bone resorption in mice, and a blocking antibody to the β-subunit of FSH has been shown to prevent bone loss (31). Furthermore, blocking FSH has recently been shown to induce thermogenic adipose tissue and reduce body fat in wild-type mice (32). Our clinical findings in women with defective FSHR function do not support the proposed therapeutic potential of FSH-blocking agents in humans. However, it remains to be investigated whether this type of FSH action in humans is conveyed through a different receptor than the FSHR.

Infertility is often the most devastating aspect of POI diagnosis. Women with FSHRO have had the chance to conceive using modified IVF and donated oocytes since the early 1990s. Early results of these treatments have been previously reported (7). Our results are in line with these and show that IVF with donated oocytes is an effective treatment in this patient group despite the severe hormonal abnormality. The rate of pregnancy complications did not differ from that in other IVF pregnancies (33). Recently, *in vitro* maturation (IVM) was utilized in treating a woman with FSHRO (34). However, the etiology in this case remains unclear, and the patient did not harbor a mutation in FSHR, as all our patients do. Nevertheless, IVM would provide an attractive option for FSHRO women, allowing them to have biologically related children.

Women with POI have reported significant levels of psychological symptoms such as grief, sadness, and diminished self-esteem (35). Depression was more common in the FSHRO women compared to the national prevalence; however, majority of the women had no psychological morbidity. A potential explanation could be that the FSHRO women were already psychologically adjusted to the POI diagnosis at the time of the current study, since due to their primary amenorrhea, their diagnoses had been made at a younger age compared with older POI patients. Additionally, all FSHRO patients have had genetic counseling to explain the genetic etiology of the POI, which may have enhanced their psychological adjustment. However, a majority of the FSHRO women were at risk or at high risk of developing sexual dysfunction. This is in line with the results of a Brazilian study on 58 POI patients, where the prevalence of sexual dysfunction was 62.1% (36). Similarly, low scores on a sexual function scale were reported in young women with spontaneous POI who received physiologic estradiol replacement (37). Women with POI should be given advice concerning the importance of HT and the role of regular sexual activity to prevent vaginal atrophy. In addition, sexual counseling should be available for this patient group.

In conclusion, our results imply that HT of twenty years is sufficient in prevention of major cardiovascular or metabolic morbidity. However, optimal bone health and bone mineral loss prevention may require early-onset and a longer period of HT, mimicking the natural fertile period. Women with FSHRO as well as other women with POI should be educated about the health effects of hypoestrogenism and encouraged to use optimal HT. In addition, sexuality and fertility are core issues for women with POI, and these should be addressed by health care personnel.

## Supplementary Material

Supplementary figure 1. Reproductive outcomes of the FSHRO cohort.

Supplementary table 1

## Declaration of interest

 The authors declare that there is no conflict of interest that could be perceived as prejudicing the impartiality of the research reported.

## Funding

This work was financially supported by the Finnish Medical Society (K L), The Sigrid Juselius Foundation (J S T), the Academy of Finland (J S T), the Helsinki University Hospital Research Fund (K L, J S T) and Finska Läkaresällskapet Fund (K A).

## Author contribution statement

J S T, K A and K L designed the study and participated in the recruitment of the FSHRO women. K L conducted practical work regarding health questionnaire, performed clinical examinations and wrote the first draft of the article. P J designed the study and acquired the data of the control group. All authors participated in data analysis, critical reading and discussion of the article and approved the final version of the manuscript to be published.
