# Supplementary figures and images for: Long-term health of women with genetic POI due to FSH-resistant ovaries
Source: Endocr Connect. 2019 Sep 9;8(10):1354–62. doi: 10.1530/EC-19-0244 (PMC6790899; doi:10.1530/EC-19-0244)

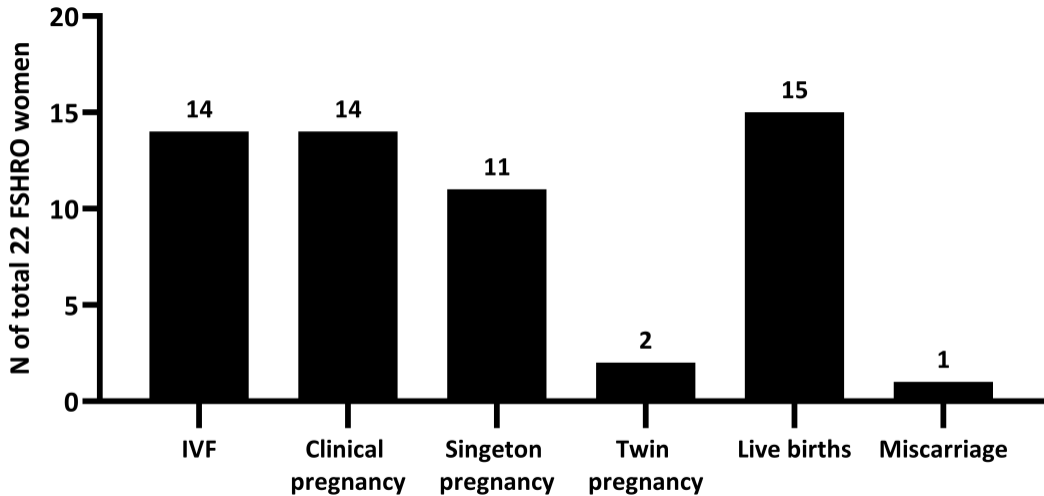

Supplement: Supplementary figure 1. Reproductive outcomes of the FSHRO cohort. [file supplementary_figure_1.pdf]
