# Supplementary material for: Long-term health of women with genetic POI due to FSH-resistant ovaries
Source: Endocr Connect. 2019 Sep 9;8(10):1354–62. doi: 10.1530/EC-19-0244 (PMC6790899; doi:10.1530/EC-19-0244)
Supplement: Supplementary table 1 [file supplementary_table_1.pdf]

Supplementary table 1

	FSHRO Q+C			FSHRO Q		
		range	±SD		range	±SD
**N**	14			8		
**Age**	49.6	28-70	11.5	49.6	30-70	15.1
**Height (m), mean**	1.63	1.54-1.71	0.1	1.61	1.56-1.70	0.1
**BMI (kg/m^2), mean**	24.8	20.6-41.3	5.5	27.3	21.9-32.9	4.0

Comparison on of the demographic characteristics of the FSHRO women who took part in both questionnaire and clinical examination (Q+C) and FSHRO women who only retuned the questionnaire (Q).
